# What caused neonatal deaths in Senegal in 2017? a secondary analysis of 2017 DHS

**DOI:** 10.11604/pamj.2020.37.268.26513

**Published:** 2020-11-25

**Authors:** Ndèye Marème Sougou, Jean Baptiste Diouf

**Affiliations:** 1 Department of Preventive Medicine and Public Health, University Cheikh Anta Diop, Dakar, Senegal,; 2 Institute of Health and Development, University Cheikh Anta Diop, Dakar, Senegal,; 3 Unité Mixte International 3189, UCAD, CNRS, Dakar, Senegal,; 4 Hospital Roi Baudoin, Ministry of Health, Dakar, Senegal

**Keywords:** Neonatal death, associated factors, Senegal

## Abstract

**Introduction:**

in Senegal, the fight for newborn and child survival is a public health priority. The aim of this study is to analyze the factors associated with neonatal deaths in Senegal in 2017.

**Methods:**

this article used data from the Senegal Demographic and Health Survey 2017. It covered 6073 children under the age of 5. The sample from the 2017 Continuous DHS is nationally representative. A bivariate analysis was conducted. The multivariate analysis was performed using STATA 15 software. Adjusted odds ratios had been calculated for variables with significant p values. The dependent variable was neonatal death.

**Results:**

a total of 6,073 children had been investigated. The neonatal death rate is 2.12%. Neonatal deaths account for 50.97% of all infant and child deaths. Newborns with a low birth weight < 2500 g are 2.3 times more likely to die with an ORaj of 2.3 [1.01-5.28]. Newborns who are considered “very small” by their mother at birth are 2.5 times more likely to die in the neonatal period ORaj=2.5 [1.04-6.04]. The last risk factor identified is birth by caesarean section (ORaj=3.97 [1.68-9.39]).

**Conclusion:**

this study concludes that low birth weight is an important risk factor for neonatal deaths in Senegal. These results suggest better management of antenatal care. However, this study showed that there was a deficit in the provision of perinatal services in Senegal. A qualitative analysis of caesarean section in the context of universal coverage could be a perspective for further reflection on improving newborn survival in Senegal.

## Introduction

Approximately 4 million neonatal deaths occur worldwide each year, three-quarters of which occur in the first week of life with a higher risk on the first day of life [1]. Fifteen thousand (15,000) children under the age of 5 died every day in 2016, 46% of them in the first 28 days of life [2, 3]. Most neonatal deaths occurred in two regions: South Asia (39%) and sub-Saharan Africa (38%). In Senegal, the fight for newborn and child survival remains a priority. Between 2010 and 2016, neonatal mortality, has dropped slowly compared to infant and child mortality, dropping from only 26.62 in 2010 to 21.82 per 1000 live births in 2016 [4]. Several studies have been done to identify risk factors for neonatal death. However, many of these studies have been conducted in hospital settings and have focused on direct causes of neonatal death such as perinatal asphyxia, neonatal infections and prematurity [5, 6].

However, population-based studies have been able to identify some of the risk factors for neonatal death [7]. These were individual factors related to the mother (absence of maternal partner, maternal age ≥35 years, multiple gestation), factors related to individual characteristics of the child (male, low and very low birth weight, weeks of gestation age ≤37) and factors related to the use of neonatal benefit services (inadequate and absent prenatal care, and cesarean delivery) [7, 8]. At the time of the Millennium Development Goals (MDGs), it appears that Senegal, like many West African countries, had not achieved the objectives concerning the reduction of infant and child mortality, and more specifically that of neonatal mortality [9]. Many regions, particularly in West Africa, have committed themselves to reducing under-five mortality rates by at least 8 per cent per year between 2015 and 2030, in order to achieve the sustainable development goal (SDG 3.2) for under-five mortality by 2030 [10]. In the same vein, Senegal has undertaken to reduce its neonatal mortality rate by 2035 [11]. This study will provide recommendations for the direction of control strategies to reduce neonatal death. The aim of this study is to analyze the factors associated with neonatal deaths in Senegal in 2017.

## Methods

**Study and sampling:** this was a descriptive cross-sectional study using DHS 2017 data. The DHS sample is representative at the national level, at the regional level, for urban and rural areas, and at the level of the 14 regions of Senegal. The DHS sample is drawn stratum by stratum. Thus, in accordance with the DHS methodology, the sample is based on a stratified, two-stage, areal sample drawn in accordance with the DHS sampling methodology [12]. For the DHS in Senegal, at the first level, the survey covers 400 clusters (Primary Survey Units UPS) which are drawn from the list of Enumeration Zones (ZD) established during the General Census of Population and Housing, Agriculture and Livestock (RGPHAE), using a systematic draw with probability proportional to size, the size of the UPS being the number of households [12]. A count of households in each of these clusters provides a list of households from which a second-stage sample of 22 households per cluster was drawn, in both urban and rural areas with an equal probability systematic draw. The file used is the Kids Recode (KR) [13]. The unit of analysis for this file is: child under 5 years of age born to a female interviewee. This file contains information on the child's pregnancy and post-natal care, as well as data on immunization, health and nutrition. Data on the mother of each of these children are also included.

**Study variables:** the dependent variable was neonatal death. This variable was created from the combination of two DHS variables: a qualitative variable that is the living or deceased status of the child and a continuous variable that is the age at death in months (imputed) [13]. The study considered explanatory variables related to individual factors, socio-demographic factors, and factors related to neonatal service provision.

**Individual factors:** these were: sex of the child; birth weight of the child in kilograms; estimated height of the child at birth (This is the number of live births during the 5 years preceding the survey distributed according to the mother's estimate of the size of the baby at the time of birth (very small, smaller than average, average or larger, don't know/unknown)); previous birth interval (months).

**Socio-demographic factors:** the socio-demographic factors were the age of the mother: Age was analyzed by 5-year age groups (15-19 years; 20-24 years; 25-29 years; 30-34 years; 35-39 years; 40-44 years; 45-49 years); Place of residence: it had been dichotomized into “urban” or “rural”; the mother's level of education; the father's level of education; the sex of the head of household; the level of household wealth : the wealth index, a measure of relative economic well-being based on household assets, was ranked by quintiles (lowest, second, middle, fourth, highest) and derived from the wealth score.

**Neonatal service delivery factors:** these were the number of prenatal visits during pregnancy, the time after delivery the postnatal check-up took place, the function of the person who made the postnatal check-up visit, the place of delivery and delivery by caesarean section.

**Statistical analysis:** the analysis was carried out using STATA/SE 15.1 software. As mentioned above in the section on data source, a two-stage sampling plan was adopted. To accommodate the multi-stage sampling design of the survey, all data were weighted to account for disproportionate sampling and non-response. In the descriptive analysis, the variables were presented in terms of frequency and percentage of data. Inter-group comparisons were made using the Chi2 test. The significance level was set at 5, and 95% confidence intervals (CIs) were used. Variables with p less than 0.25 in the bivariate analysis were selected for multivariate analysis [14]. To assess the factors associated with neonatal death, a multivariate logistic analysis was performed to account for the effect of confounding factors. Adjusted odds ratios (ORa) were calculated with their 95% confidence intervals. To handle complex sampling (multistage sampling, weighting and stratification), the identification variables for weights, strata and primary sampling units (PSUs) were defined before using the SVY (STATA survey prefix).

**Ethical approval:** this study is a secondary analysis of DHS data from Senegal in 2017. The 2017 Demographic and Health Survey (DHS) in Senegal has been approved by the National Ethics Committee (CNERS). The survey has also been approved by the Ethics Committee (Institutional Review Board) of the ICF. The informed consent obtained from all participants was written.

## Results

**Socio-demographic characteristics:** a total of 6,073 children had been investigated. Individual characteristics of children under 5 years of age in Senegal. Forty-eight-point ninety-three percent (48.93%) of the children were female. Five-point six point seven (5.67%) of the children had low birth weight. Zero-point thirty-one percent (0.31%) had a very low birth weight <1500g. Fourteen-point forty-five percent (14.45%) of children had a reproductive interval prior to birth < 24 months (Table 1).

**Table 1:** description of the individual characteristics of the children

	Freq absolute	Percentage %
**Child Sex (n=6073)**		
Male	3101	51.07
Female	2972	48.93
**Birth weights (n=5860)**		
Weight ≥ 2500g	5509	94.02
[1500-2500 g]	332	5.67
[500-1500g]	18	0.31
**Size of child at birth**		
Very large	631	10.38
larger than average	861	14.18
Average	2572	42.35
Smaller than average	1055	17.37
Very small	940	15.47
Don´t know	15	0.24
**Precedent reproductive interval less than 24 months (n=4684)**		
No	4007	85.55
Yes	678	14.45
**Distribution of children according to the probability of death in the neonatal period in Senegal (n=6073)**		
Survived more than 1 month	5944	97.88
Deceased before 1 month	129	2.12

**Socio-demographic characteristics of children under 5 years of age in Senegal:** children living in rural areas was 63.22%. Most mothers and their partner/husband were uneducated with 64.63% and 68.40% respectively. Only 25.97% of the heads of households were women (Table 2).

**Table 2:** distribution of children by socio-demographic characteristics

	Freq absolute	Percentage %
**Place of residence**		
Urban	2233	36.78
Rural	3839	63.22
**Household wealth level**		
Poorest	1537	25.30
Poor	1377	22.68
Middle	1137	18.72
Richer	1107	18.22
Richest	915	15.07
**Mother´s age (5-year-old group)**		
15-19	287	4.72
20-24	1148	18.90
25-29	1701	28.00
30-34	1352	22.26
35-39	930	15.31
40-44	514	8.46
45-49	142	2.34
**Mother´s level of education**		
No education	3925	64.63
Primary	1315	21.65
Secondary	724	11.93
Higher	109	1.79
**Level of education of husband/partner**		
No education	4096	68.40
Primary	567	9.47
Secondary	524	8.75
Higher	215	3.58
Don´t know	587	9.80
**Religion**		
Muslim	5896	97.09
Christian	154	2.54
Animist	23	0.38
**Sex of the head of household**		
Male	4495	74.03
Female	1577	25.97

**Characteristics of neonatal service provision in Senegal:** fourteen-point sixty-nine percent (14.69%) of children had received postnatal care by unqualified health personnel. Out of 1038 mothers, only 44.70 per cent of respondents reported receiving NCPC after their birth, including at home. Twenty-four decimal zero nine percent (24.09%) of children were born at home (22.12% in the mother's home). Five-point five percent (5.5%) of children were born by caesarean section (Table 3). Four-point seventy-four percent (4.74%) of mothers did not have prenatal visits (Table 3). Seventy-eight ninety-six per cent (68.96%) of children did not receive essential health care from the first hour of birth (Table 3).

**Table 3:** distribution of children by characteristics of neonatal service provision

	Freq Absolute	Percentage %
**Person who performed the postnatal check-up**		
Doctor	138	3.82
Midwife	1954	54.23
Nurse	883	24.51
Trained traditional birth attendant	529	14.69
Traditional birth attendant	24	0.66
Other	75	2.09
**Post-natal consultation after childbirth including at home (n=1038)**		
No	574	55.30
Yes	464	44.70
**Place of delivery n=6073**		
Personal residence of the birth	1343	22.12
Another home	120	1.97
Public Hospital	435	7.16
Health Centre/Public Maternity Hospital	1117	18.40
Health post	2604	42.89
Health case	187	3.09
Other public health structures	22	0.36
Hospital/private clinic	237	3.91
Other private health structure	5	0.08
Other	2	0.04
**Cesarean delivery (n=6005)**		
No	5672	94.45
Yes	333	5.55
**Number of pre-natal visits carried out(n=4575)**		
No prenatal visits	217	4.74
1	227	4.96
2	587	12.83
3	1199	26.21
4	1695	37.05
More than 4 antenatal visits	650	14.21
**Newborn care from the first hour of birth (n=6073)**		
No	4188	68.96
Yes	1885	31.04

**Neonatal death rate:** the neonatal death rate is 2.12% (Table 1). Neonatal deaths account for 50.97% of all infant and child deaths.

**Multivariate analysis; factors associated with neonatal death in Senegal:** newborns with a low birth weight <2500 g are 2.3 times more likely to die with an ORaj of 2.3 [1.01-5.28]. Newborns who are considered “very small” by their mothers at birth are 2.5 times more likely to die in the neonatal period ORaj=2.5 [1.04-6.04]. The last risk factor identified is birth by caesarean section of the child (ORaj=3.97 [1.68-9.39]) (Table 4).

**Table 4:** multivariate analysis factors associated with neonatal deaths

Neonatal death	ajOR	[95% CI]	p
**Sex**			
Male	1		
Female	0.89	[0.55-1.46]	0.656
**Birth weight < 2500 g**			
No	1		
Yes	2.30	[1.01-5.28]	0.049
**Estimated size of the newborn by the mother at birth**			
Very large	1		
larger than average	1.46	[0.55-3.92]	0.448
Average	0.78	[0.32-1.90]	0.579
Smaller than average	1.35	[0.50-3.66]	0.56
Very small	2.50	[1.04-6.04]	0.042
Don´t know	8.56	[0.84-86.94]	0.069
**Place of residence**			
Urban	1		
Rural	1.52	[0.68-3.38]	0.305
**Level of household wealth**			
Poorest			
Poor	0.94	[0.52-1.67]	0.825
Middle	0.96	[0.45-2.06]	0.923
Rich	0.25	[0.05-1.19]	0.082
Richest	0.40	[0.08-2.07]	0.273
**Level of education of husband/partner**			
No education	1		
Primary	1.37	[0.54-3.50]	0.511
Secondary	1.47	[0.47-4.58]	0.511
Higher	1.51	[0.19-12.26]	0.701
Don´t know	1.03	[0.39-2.67]	0.957
**Mother's level of education**			
No education	1		
Primary	0.93	[0.45-1.92]	0.84
Secondary	0.75	[0.21-2.72]	0.664
Higher	1.00		
**Sex of the head of household**			
Male	1		
Female	0.68	[0.34-1.37]	0.277
**Previous Reproductive Interval < 2 years**			
No	1		
Yes	1.46	[0.78-2.74]	0.234
**Birth by Caesarean section**			
No	1		
yes	3.97	[1.68-9.39]	0.002
**Number of antenatal visits performed**			
0	1		
1	1.09	[0.32-3.75]	0.889
2	1.04	[0.36-2.98]	0.942
3	0.74	[0.26-2.09]	0.573
4	0.74	[0.26-2.06]	0.561
More than 4 antenatal visits	0.38	[0.10-1.50]	0.168

## Discussion

**Neonatal death:** mortality among newborns declines more slowly (2.9% per year) than among children aged 1-59 months (4.9%). In several countries around the world, it has tended to increase from 37.6% to 43.9% between 2000 and 2013 [15]. This result explains the need to reduce neonatal mortality in order to reduce child mortality. This study found a neonatal mortality rate of 2.12% (21.2/1000 live births). These figures are similar to those found in other countries in the West African sub-region. Thus in the Gambia, the neonatal mortality rate is 21/1000 live births [16]. In Burkina Faso, neonatal mortality rates are 46.3/1000 live births. In general, neonatal mortality rates are high overall in West Africa and can exceed rates of 50/1000 live births in some parts of West Africa [10, 17]. The share of neonatal deaths in under-five mortality remains high. Neonatal deaths account for 50.97% of under-five deaths in this study. This is the case in many African countries where more than half of under-five deaths occur in the neonatal period [18]. In the Gambia, the share of neonatal deaths accounts for 40% of the under-five population [16]. This significant contribution of neonatal deaths to infant and child mortality reinforces the interest that should be focused on this issue as part of the fight against infant and child mortality in West Africa and Senegal in particular.

**Factors associated with neonatal death:** this study showed a low birth weight rate of 5.92%. Newborns with a low birth weight < 2500g were 2.3 times more likely to die with an ORaj of 2.3 [1.01-5.28]. Studies have shown that low birth weight, particularly very low birth weight, is a major determinant of the magnitude of high neonatal mortality rates [19, 20]. The role of low birth weight in the occurrence of neonatal deaths has been described in the literature as follows [21]. The mother's recall of the birth events can thus be considered an important source of information in the diagnosis of health problems in children under 5 years of age [22]. This study showed that newborns who were considered “very small” by their mothers at birth were 2.5 times more likely to die in the neonatal period ORaj=2.5 [1.04-6.04]. This estimate was based on the judgement of mothers at the birth of their child. Maternally reported information collected through national household surveys, such as Demographic and Health Surveys (DHS) and Multiple Indicator Cluster Surveys (MICS), is often the only source of demographic data available on birth weight and preterm birth indicators in low-income countries [23]. As part of these approaches, mothers are asked to recall events related to the birth of their child that may have taken place up to five years prior to the administration of the survey [23]. These results contribute to the reflection on the importance of local denominations that can serve as a basis for the promotion of neonatal health in the African context where a large number of deliveries and post-natal care are still carried out outside health facilities or by unqualified personnel [24].

This study was able to identify caesarean birth as a factor in neonatal death (ORaj=3.97 [1.68-9.39]). The practice of caesarean section is in increasing evolution in the world although the evolution of this practice in sub-Saharan Africa is still timid [25]. In Senegal, our study showed caesarean delivery rates of 5.55%. This is at the limit of the rates recommended by the WHO guidelines (5-15%) [26]. However, our study was able to establish that children born by caesarean section in Senegal were 3.97 times more likely to die during their first month of life. The reasons could be related to the quality of the C-sections performed. C-section is an essential maternal health service. Its role in labor and delivery care in low- and middle-income countries is complex; in many resource-poor settings it is underutilized in the neediest populations and over utilized by the least needy, without clear methods to ensure universal access [27]. Studies have shown that there are no statistically significant differences among the different types of caesarean section procedures that exist [27, 28]. Thus, complications from caesarean section may not be due to the caesarean section itself but rather to the caesarean section procedure [28]. Several studies have highlighted the complications of caesarean sections in sub-Saharan African countries, among which infections have been cited as among the most important [29]. It should be noted that caesarean section in excellent condition would allow high rates of up to about 19 per 100 live births to be associated with lower neonatal mortality among WHO member states [30].

Thus, the provision of perinatal services needs to be examined. In Africa, the number of active health workers does not yet meet the demand for coverage of the health needs of the populations [24]. Consequently, it would be possible to note an inadequacy in the quality of care for newborns at birth. The study showed that 14.69 per cent of children did not receive post-natal care by qualified health personnel. Also, only 44.70 per cent said they had received post-natal visit after delivery, while 24.09 per cent of children were born at home (with 22.12 per cent in the mother's home and the rest in another home in the community). Sixty-eight-point ninety-six percent (68.96%) of the children had not received essential post-natal care from the first hour of birth. Early postnatal care is essential for the promotion of healthy practices in the home, including exclusive breastfeeding, and is crucial for the health and survival of children. However, despite the benefits of postnatal care, most newborns and mothers do not benefit from the provision of this care by a skilled health worker during the critical first few days after delivery [31].

**Limitations:** this study has certain limitations. In particular, the analyses used cross-sectional data, so that only associations and no causal relationships were established.This study could be complemented by a qualitative study to understand the contextual mechanisms and effects of health interventions to combat neonatal mortality in Senegal.

## Conclusion

On the basis of the findings of this study, it is important to note that neonatal mortality remains high in Senegal and accounts for a significant proportion of infant and child mortality. The study showed that the factors associated with neonatal mortality in Senegal were low birth weight, estimation of the newborn's body weight by the mother and birth by caesarean section. The identification of factors associated with low birth weight could be the subject of further study. However, this study may have shown gaps in the provision of perinatal services (antenatal and postnatal visit). Furthermore, the mother's estimation of the newborn's corpulence, which has been identified as an associated factor, lays the foundations for thinking about health promotion in the sense of using mothers' perceptions and representations of their newborns. This conclusion ends with a reflection on caesarean sections in Senegal, which despite a still low rate of 5.5% compared to recommended rates, is one of the risk factors for neonatal death, in contrast to its primary function of contributing to maternal and neonatal survival. Thus, the technical conditions for performing caesarean sections and the factors of their social acceptability could be better examined in Senegal in the context of the fight against neonatal mortality. An evaluation of the free caesarian program in Senegal could provide some answers regarding the quality of the caesarian in this country.

### What is known about this topic

- Neonatal deaths remain high in Senegal and account for a large proportion of infant and child mortality;
- Perinatal services are still insufficient in Senegal.

### What this study adds

- Mother's estimation of the newborn's corpulence, which has been identified as an associated factor, lays the foundations for thinking about health promotion in the sense of using mothers' perceptions and representations of their newborns;
- An evaluation of the quality of caesarean sections performed in Senegal would restore its life-saving role and eliminate it from the list of factors contributing to neonatal death in Senegal.
